# A Combined Small-Angle X-ray and Neutron Scattering Study of the Structure of Purified Soluble Gastrointestinal Mucins

**DOI:** 10.1002/bip.22523

**Published:** 2014-07-11

**Authors:** Pantelis Georgiades, Emanuela di Cola, Richard K Heenan, Paul D A Pudney, David J Thornton, Thomas A Waigh

**Affiliations:** 1 Biological Physics, Department of Physics and Astronomy, University of ManchesterManchester, M60 1QD, UK; 2 Faculty of Life Sciences, University of Manchester, Michael Smith BuildingOxford Road, Manchester, M13 9PT, UK; 3 European Synchrotron Radiation Facility6 rue Jules Horowitz, BP-220 Grenoble, Cedex, 38043, France; 4 ISIS Facility, Rutherford-Appleton LaboratoryChilton, Didcot, OX11 0QX, UK; 5 Unilever Discover, Colworth ParkSharnbrook, Bedfordshire, MK44 1LQ, UK; 6 Photon Science Institute, University of ManchesterOxford Road, Manchester, M60 1QD, UK

**Keywords:** mucin, gastric, intestinal, SANS, SAXS, DLS

## Abstract

The structures of purified soluble porcine gastric (Muc5ac) and duodenal (Muc2) mucin solutions at neutral and acidic pH were examined using small-angle X-ray scattering and small-angle neutron scattering experiments. We provide evidence for the morphology of the network above the semidilute overlap concentration and above the entanglement concentration. Furthermore, we investigated the gelation of both types of mucin solutions in response to a reduction in pH, where we observed the formation of large-scale heterogeneities within the polymer solutions, typical of microphase-separated gels. The concentration dependence of the inhomogeneity length scale (Ξ) and the amplitude of the excess scattering intensity [I_ex_(0)] are consistent with previously studied gelled synthetic polymeric systems. The persistence lengths of the chains were found to be similar for both Muc5ac and Muc2 from Kratky plots of the neutron data (8 ± 2 nm). © 2014 Wiley Periodicals, Inc. Biopolymers 101: 1154–1164, 2014.

## INTRODUCTION

The O-linked glycoproteins known as mucins act as the building blocks of the mucus layer, the viscoelastic fluid that covers the epithelium of various organs. This mucus layer acts as the first line of defense against external risks, including pathogens and inhaled or ingested foreign particles, while ensuring the lubrication of epithelial surfaces.^1^,^2^ Moreover, in the stomach, the role of the mucus barrier is to protect the epithelium from its hostile environment, especially during active digestion when the pH of the secreted gastric juices can be as low as pH 1 to assist with protein digestion.^3^ Furthermore, the mucus layer in the small intestine acts as a host to bacteria responsible for the breakdown of ingested food to nutrients, while allowing the diffusion of these nutrients through it for them to be absorbed by the body.^4^ Large-scale proliferation of huge quantities of intestinal bacteria can be detrimental for human health as observed in small intestinal bacteria overgrowth syndrome.^4^,^5^

Mucus consists mainly of water (∼95%), immunoglobulin, cholesterol, lipids, inorganic salts, proteins, and the aforementioned glycoproteins called mucins (2–3% w/w). Polymeric mucins are very large (*M*_w_ ranges from 2.5 MDa to tens of MDa for secreted oligomers) heavily glycosylated proteins, which form the structural basis of the mucus layer. There are two families of mucins, the polymerizing and nonpolymerizing secreted mucins and the cell-tethered mucins, which share many common features.^1^ The weight of both types of mucins is accounted for primarily (approximately 70–80%) by carbohydrates, mainly *N*-acetylgalactosamine, *N*-acetylglucosamine, fucose, galactose, and sialic acid. They are characterized by at least one large region of the polypeptide that is rich in proline, threonine, and serine residues, known as the PTS domain. These domains act as sites for addition of O-linked glycans, which results in extensive glycosylation, thus the molecular weight of the high proportion of the proteins is accounted for by carbohydrates.^1^,^2^,^6^ A shared feature amongst polymerizing secreted mucins (the focus of the current work) are cysteine-rich domains (D-domains) at their N- and C-termini, which facilitate the formation of disulfide bonds between mucin monomers during synthesis, thus allowing the formation of linear or branched polymeric aggregates and the polymeric semidilute mesh when the aggregates overlap. It is mucin's large size and complex nature though, that has made techniques commonly used for the characterisation of proteins, such as NMR and crystallography, inadequate.^2^,^6^–^8^ There are still many unanswered structural questions with respect to all of the different types of mucin. Recently, an asymmetric dumbbell structure has been postulated for Muc5ac (and it may be observed in all the gelling mucins due to similarly arranged domain sequences known from genetic analysis)^9^; however, this model still requires additional evidence before it is fully accepted. Many previous studies have been hampered by a lack of chemically noninvasive preparation techniques for mucins. Samples typically used (commercially available Sigma-Aldrich and Orthana mucin) lack the associating ability of in vivo mucin and do not form gels when the pH is dropped. Additionally, the purification of mucins usually involves the use of chaotropic agents, which disrupt noncovalent bonding between the various components of mucus, and the mucins are removed with centrifugation or chromatography. For the native structure and folding of the proteins to be preserved, the use of reducing and chaotropic agents needs to be omitted, which reduces the yield of purified mucins, as these conditions exclude “insoluble” Muc2 mucin complexes that require reduction of disulfide bonds to bring them into solution.^10^ In the current structural study, we examined specimens that do exhibit such an associative ability, but do not contain insoluble mucin complexes.^11^,^12^

Recent work with AFM has pointed to a branched trigonal structure with little symmetry for Muc2 gels that were prepared in denaturing conditions (GuCl) and dried onto mica surfaces, with 20–200 nm distances between branch points.^13^ Negative-stain TEM has provided more persuasive images of Muc2 sheets with almost perfect hexagonal symmetry and with 15 nm distances between branch points.^14^ Thus, the model put forward by both studies is broadly that of elastic two-dimensional membrane layers of Muc2 mucin that stack on one another. This layered model is by no means well established^15^,^16^ and does not explain the rapid swelling of mucin gels when exposed to water,^16^ as the lamellar structure is only able to expand in one dimension (the expansion would be reduced by a cube root function when compared with a standard polyelectrolyte gel, which is not observed in experiments). A more likely possibility is that the hexagonal structure observed in TEM images^13^ exists in mucin storage granules when mucin is combined with calcium, but may not be the dominant component that occurs in mucus layers in the intestines when they expand in volume by many orders of magnitude into random mucus polyelectrolyte gels. Implications for the hexagonal membrane model are discussed in more detail with respect to the results later in the article.

The scaling of viscoelasticity of mucins with concentration and the effects of low pH was recently investigated by our group by means of particle-tracking microrheology (PTM), which concluded that three distinct concentration regimes exist for the dynamics of gelling mucins.^11^ At low concentrations, mucin oligomers diffuse in solution with little interaction between themselves. Above a critical concentration, c*, mucin oligomers start to interact with each other and overlap in a semidilute network. The oligomers form linear aggregates, and the viscosity (*η*) of the semidilute solution scales with concentration (*c*) as *η* ∼ *c*^1/2^, typical of overlapping linear flexible polyelectrolytes. Above a second critical concentration, the entanglement concentration (*c*_e_), the protein backbone and the side-chain carbohydrates reptate and the viscosity scales as a strong function of the mucin concentration (*c*), as *η* ∼ *c*^3.9 ± 0.38^ for Muc5ac, the mucin gene predominantly found in stomach mucus, and *η* ∼ *c*^5.1 ± 0.8^ for Muc2, the mucin gene found in intestinal mucus. Furthermore, both types of mucins undergo a sol–gel transition when the pH of the solution was reduced to pH 1.4 using HCl.^11^

In this report, we expand on our previous PTM studies of biochemically well-characterized pig gastrointestinal mucins to extract the structural characteristics of mucins in the semidilute regime. A combination of scattering techniques allowed us to probe a wide range of length scales within the mucin solutions. Small-angle X-ray scattering (SAXS) experiments with Muc5ac solutions and small-angle neutron scattering (SANS) experiments with Muc5ac and Muc2 solutions provide insights about the internal organization of mucins in the polymer mesh, as well as the aggregate sizes.

## RESULTS

The purified mucin samples used in the following experiments were carefully isolated from crude mucus scrapings at low temperatures and in the presence of proteinase inhibitors and sodium azide to avoid cleavage of the macromolecules that were soluble under the extraction conditions. The molecular weight of the two mucin preparations was measured using standard multiangle light scattering techniques and was found to be in the expected range for secreted polymerizing mucins (both types of mucins exhibit *M*_w_ distributions ranging from 2 to tens of MDa; Supporting Information Figure S3). Additionally, the mucins used in this study were previously demonstrated in PTM studies to possess the associative properties expected from these biopolymers.^11^

### Small-Angle X-ray Scattering Experiments

**FIGURE 1:**
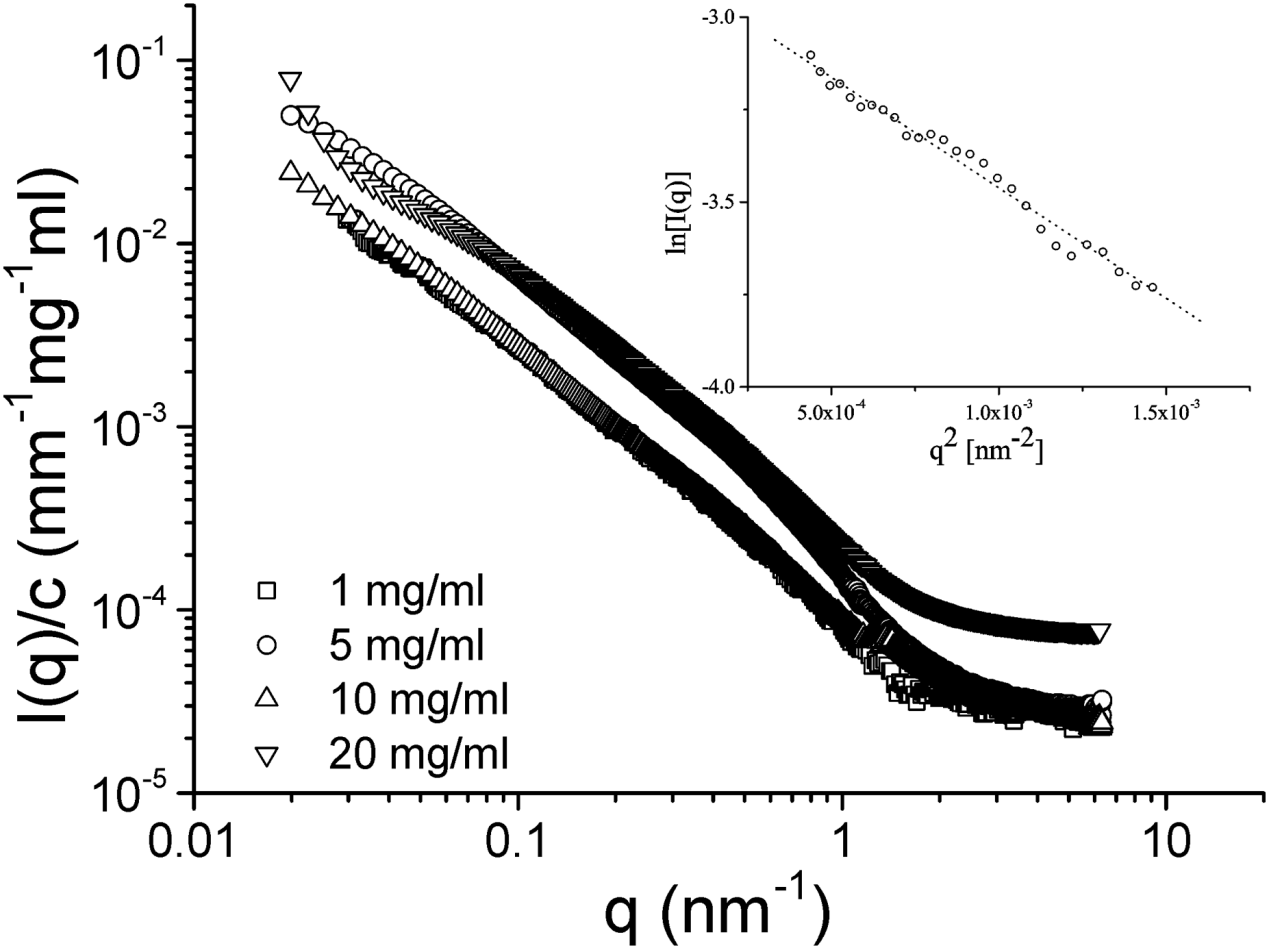
Reduced intensity (*I*/*c*) as a function of momentum transfer (*q*) for Muc5ac solutions at pH 7, obtained from small-angle X-ray scattering experiments. The inset shows a Guinier plot from a 1 mg/ml Muc5ac solution.

The internal structure of the mucin network was examined using small-angle X-ray scattering (SAXS). Figure 1 shows the concentration-normalized scattering profiles obtained from Muc5ac solutions of varying concentration at pH 7. All the concentrations examined produced scattering profiles with comparable Porod exponents in the intermediate *q*-range, as shown by the normalized scattering curves. By plotting ln[*I*(*q*)] against *q*^2^ (Guinier plot) for 1 mg/ml, we were able to calculate the radius of gyration of Muc5ac mucin at low concentrations to be 42 ± 4 nm.

**FIGURE 2:**
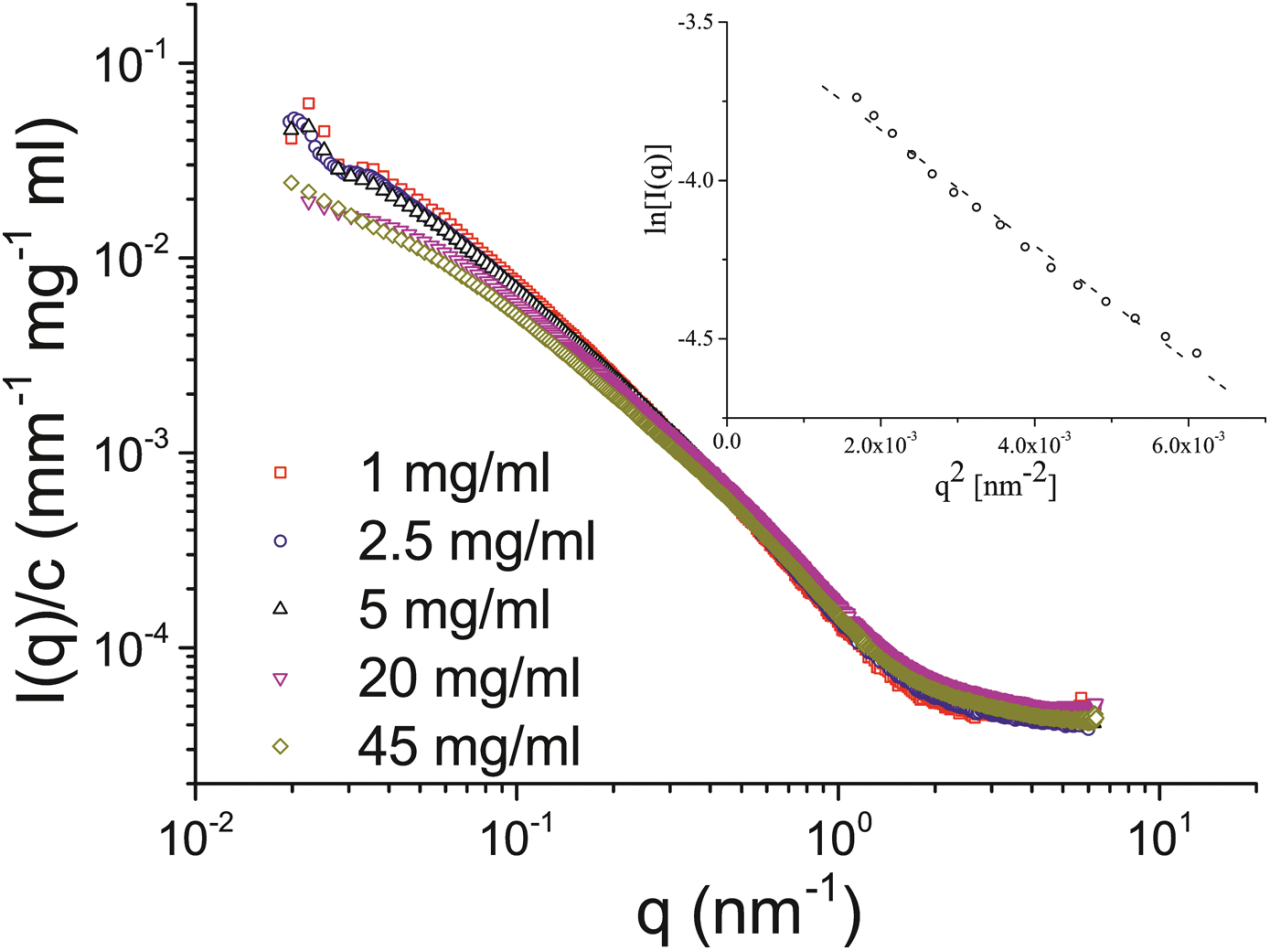
The concentration-normalized scattering profiles obtained from Muc5ac solutions at pH 3.5, the pH boundary at which gelation occurs macroscopically. The inset shows the Guinier plot of the scattering profile obtained from a 1 mg/ml solution of Muc5ac, from which the radius of gyration of the molecule was determined. All the normalized scattering profiles coincide at pH 3.5.

Figure 2 shows the concentration-normalized scattering profiles as obtained from SAXS experiments using various concentrations of Muc5ac solutions at pH 3.5, the pH boundary at which gelation occurs in gastric mucin.^2^,^12^ The absolute scattering intensity at low *q* is larger at higher concentrations when compared with the neutral pH solutions, a sign of the formation of large structures within the solution. Furthermore, the radius of gyration of the molecule at pH 3.5 for 1 mg/ml (low concentrations) was found to be 23 ± 3 nm, from Guinier analysis of the low-concentration scattering profiles, lower than the value at pH 7.

### Small-Angle Neutron Scattering Experiments

In addition to the X-ray scattering experiments, the structures of Muc5ac and Muc2 in solution were also investigated using small-angle neutron scattering (SANS). SANS experiments involve a reduction in radiation damage when compared with X-rays and thus do not require the use of a flow cell. Solutions of varying mucin concentration were examined at neutral pH (pH 7) and pH 1.4.

**FIGURE 3:**
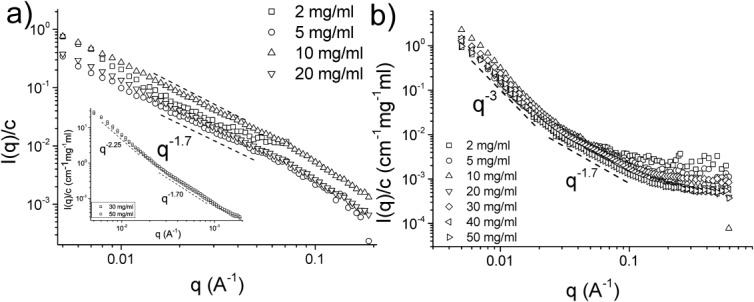
The concentration-normalized scattering profiles obtained from SANS experiments using Muc5ac solutions at (a) pH 7 and (b) pH 1.4. In pH 7 conditions, up to a concentration of 20 mg/ml, the scattering profiles follow a universal power law of *I*(*q*) ∼ *q*^−1.7^, whereas at 30 mg/ml and above there are two distinct power laws observed, *I*(*q*) ∼ *q*^−2.25^ followed by *I*(*q*) ∼ *q*^−1.7^, shown in the inset. In acidic conditions, two distinct power laws are observed, *I*(*q*) ∼ *q*^−3^ up to *q* ∼ 0.02 Å^−1^ and *I*(*q*) ∼ *q*^−1.7^ above that. The increased scattering intensity at high *q* of the 2 mg/ml solution is due to excess hydrogen being present, which decreases the contrast between the scatterer and the solvent. Furthermore, the solutions at pH 7 produced data with lower SNR, thus the accessible *q*-range was smaller when compared with the acidic solutions.

The reduced concentration-normalized scattering curves obtained from Muc5ac solutions at pH 7 and pH 1.4 are shown in Figure 3. The structural complexity of the mucins could only be described by simple minimal models for the scattering data, thus the analysis was restricted to gaining insights regarding the structures and interfaces within the solution by using robust and well-established means, such as the construction of Porod plots to calculate the various power laws which describe the scattering curves. At pH 7, the scattering profiles of the various concentrations of Muc5ac up to 20 mg/ml exhibit a universal Porod exponent equal to ∼1.7 across the whole *q*-range examined. At concentrations of 30 and 50 mg/ml, there are two distinct power laws observed in the scattering profiles, that is, a *I*(*q*) ∼ *q*^−2.25^ in the low-*q* regime and *I*(*q*) ∼ *q*^−1.7^ further on. The transition occurs at *q* ∼ 0.02 Å, which corresponds to a length scale of ∼30 nm in real space (Figure 3a). At pH 1.4, there is a significant increase in scattering intensity in the low-*q* regime, expected from solutions that have undergone a sol–gel transition. The concentration-normalized scattering profiles coincide at pH 1.4, with a Porod exponent of ∼3 in the low-*q* regime and ∼1.7 at higher *q*. The transition between the two regimes again occurs at *q* ∼ 0.02 Å.

**FIGURE 4:**
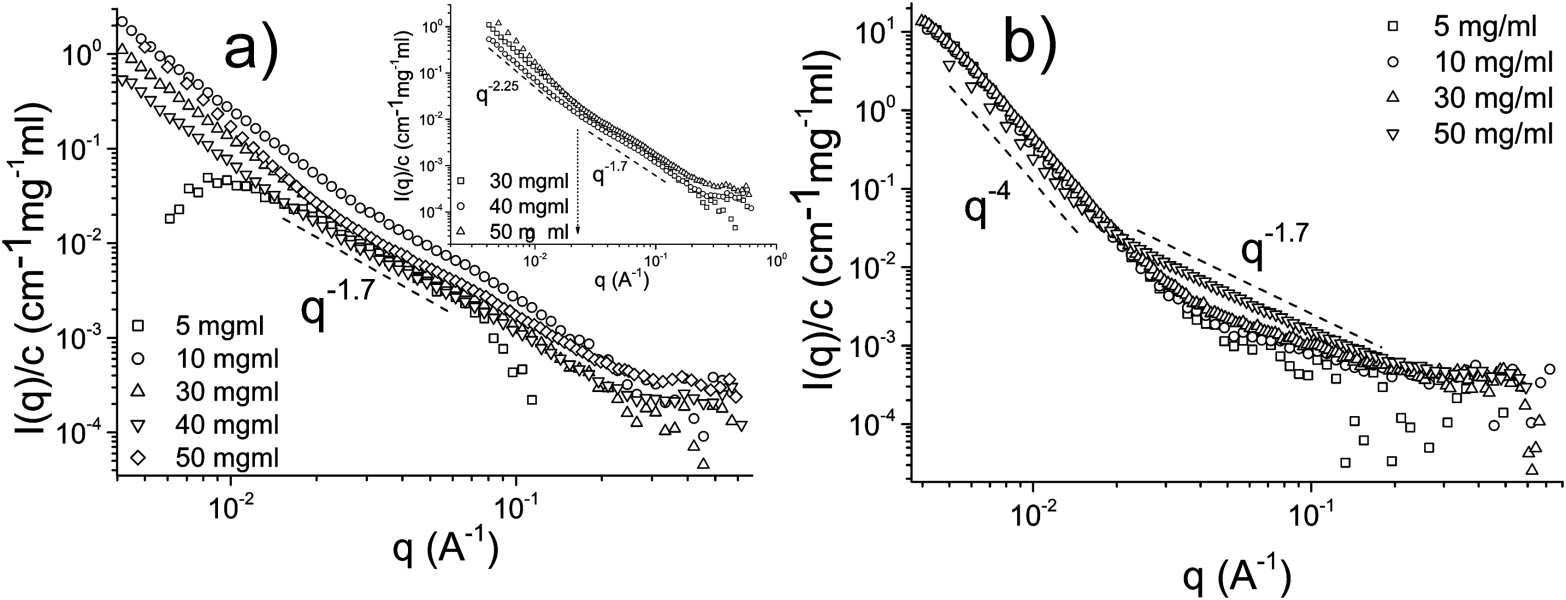
The concentration-normalized SANS scattering profiles obtained from Muc2 solutions at pH 7 and pH 1.4. (a) The scattering profiles of pH 7 Muc2 solutions. Above 30 mg/ml, there are two power laws present in the data, that is, *I*(*q*) ∼ *q*^−2.25^ at low *q* and *I*(*q*) ∼ *q*^−1.7^ in the intermediate *q*-range, as shown in the inset. The crossover occurs at *q* ∼ 0.02–0.03 Å^−1^. (b) The normalized scattering profiles of Muc2 solutions at pH 1.4. There is a large increase in intensity at low *q*, an indication of the formation of higher order structures within the solution, which exhibit a fractal dimension of ∼4. In the *q* regime above *q* ∼ 0.02, there is a universal *I*(*q*) ∼ *q*^−1.7^ power law observed.

The concentration-normalized scattering profiles from Muc2 solutions at pH 7 and pH 1.4 are shown in Figure 4. As with Muc5ac, up to a concentration of 30 mg/ml, the scattering profiles have an exponent of ∼1.7 throughout the examined *q*-range, whereas at 30 mg/ml and above, there are two distinct regimes observed (inset of Figure 4a). In the low-*q* regime and up to *q* ∼ 0.02 Å, an exponent of ∼2.25 was observed, whereas at higher *q*, an exponent of ∼1.7 was observed. For the scattering profiles acquired from the acidic solutions, there are two distinct regimes observed and a considerable increase in scattering intensity in the low-*q* regime, which indicates the formation of higher order structures within the sample. As shown in Figure 4b, there was a Porod exponent ∼4 in the low-*q* regime, whereas at the transition point at *q* ∼ 0.02 Å, there was a Porod exponent of approximately 1.4–1.7. The power law exponents observed in all the scattering profiles are presented in Table 1.

**Table 1:** The Power Law-Fit Exponents Extracted from SANS Experiments with Muc5ac and Muc2 Mucin Solutions at pH 7 and 1.4

Sample	High-*q* exponent (Muc5ac)	Intermediate-*q* exponent (Muc5ac)	High-*q* exponent (Muc2)	Intermediate-*q* exponent (Muc2)
2 mg/ml (pH 7)	1.75 ± 0.04	—	N/A	—
5 mg/ml (pH 7)	1.71 ± 0.03	—	1.64 ± 0.03	—
10 mg/ml (pH 7)	1.68 ± 0.02	—	1.63 ± 0.02	—
30 mg/ml (pH 7)	2.23 ± 0.03	1.68 ± 0.01	2.30 ± 0.01	1.73 ± 0.02
40 mg/ml (pH 7)	N/A	—	2.12 ± 0.02	1.63 ±.02
50 mg/ml (pH 7)	2.24 ± 0.03	1.70 ± 0.02	2.48 ± 0.03	1.63 ± 0.01
2 mg/ml (pH 1.4)	2.25 ± 0.06	—	N/A	—
5 mg/ml (pH 1.4)	3.16 ± 0.08	1.60 ± 0.05	4.12 ± 0.06	1.74 ± 0.16
10 mg/ml (pH 1.4)	2.97 ± 0.04	1.48 ± 0.02	4.05 ± 0.03	1.44 ± 0.09
20 mg/ml (pH 1.4)	3.08 ± 0.06	1.63 ± 0.02	N/A	—
30 mg/ml (pH 1.4)	2.94 ± 0.04	1.70 ± 0.03	4.05 ± 0.01	1.68 ± 0.05
40 mg/ml (pH 1.4)	3.07 ± 0.06	1.60 ± 0.02	N/A	—
50 mg/ml (pH 1.4)	3.02 ± 0.03	1.71 ± 0.02	3.94 ± 0.06	1.67 ± 0.02

Both mucin molecules have similar scattering at pH 7, with a general *I*(*q*) ∼ *q*^−1.7^ power law throughout the semidilute unentangled regime and two distinct regimes above that, *I*(*q*) ∼ *q*^−2.25^ up to *q* ∼ 0.02 Å and *I*(*q*) ∼ *q*^−1.7^ further on. The most noticeable difference between the two mucin types are the Porod exponents in the low-*q* regime obtained from acidic solutions, which for Muc5ac was found to be ∼3, whereas for Muc2 solutions was found to be ∼4, possibly indicating a more complete nanophase gelation transition for Muc2 mucin solutions on reduction of the pH.

**FIGURE 5:**
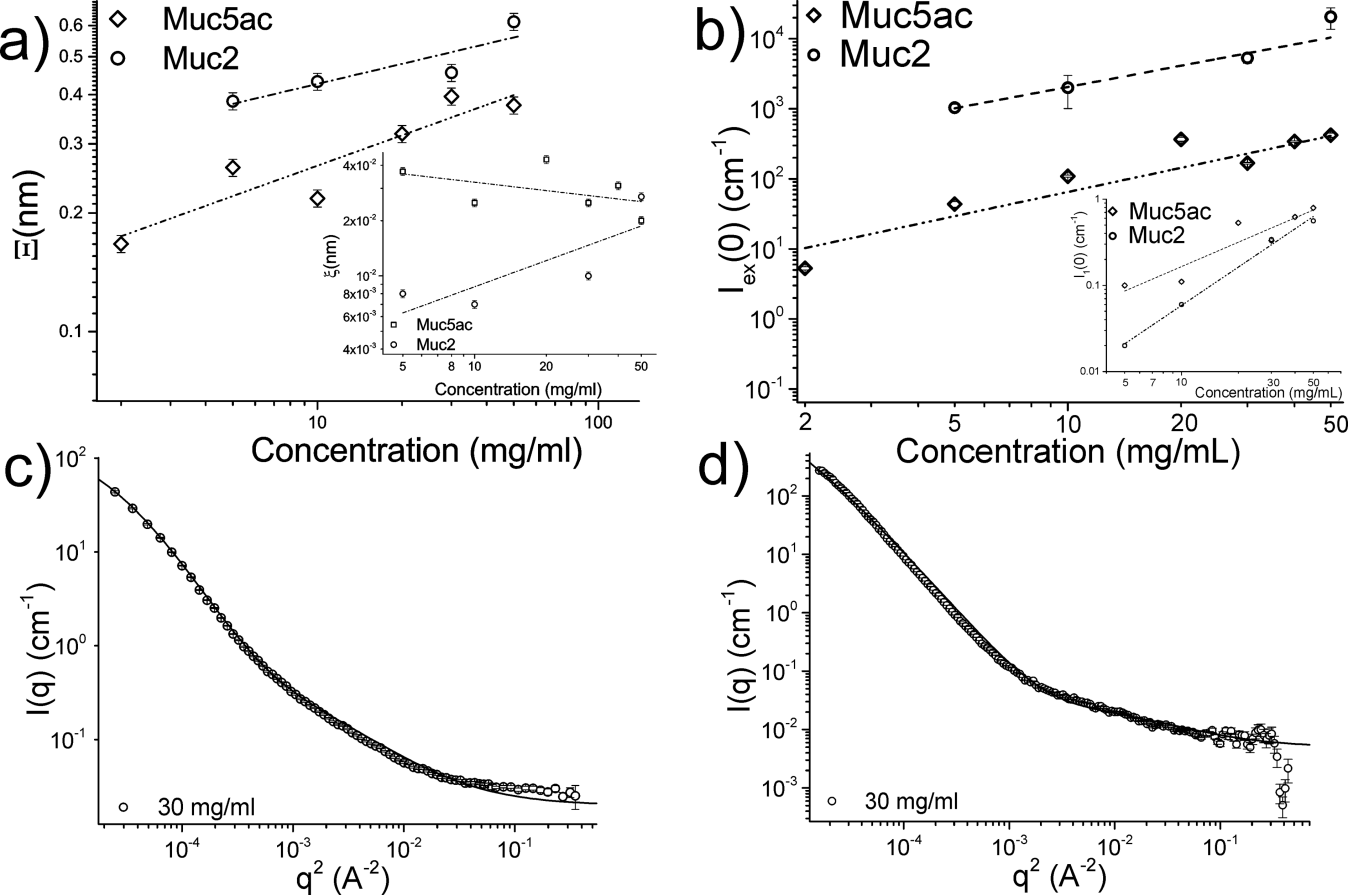
(a) The average thickness of the micro/nano phase separation aggregate (Ξ), the semidilute correlation length (*ξ*, inset) and (b) the zero-*q* excess scattering extrapolated intensity [*I*_ex_(0), *I*_1_(0)] for Muc5ac and Muc2 solutions at pH 1.4. as a function of mucin concentration. Debye-Bueche function fits [Eq. (2)] of SANS data for 30 mg/ml (c) Muc5ac and (d) Muc2 solutions, which were used to describe the micro/nano phase separated system after the reduction of the pH. As expected, Ξ, *I*_ex_(0) and *I*_1_(0) are monotonically increasing with concentration, whereas *ξ* was found to be unchanged as a function of concentration and unchanged from the corresponding solutions at neutral pH.

The semidilute correlation length (*ξ*) and the zero-*q* extrapolated intensity were calculated from the linear part of the 1/*I*(*q*) against *q*^2^ plot by fitting the Ornstein-Zernicke function, as shown in Supporting Information Figure S2 [Eq. (1)]. *I*_1_(0) is observed to monotonically increase with concentration, as expected, whereas *ξ* appears to remain constant throughout the concentration range examined, at ∼1.4 nm for Muc5ac solutions and ∼1 nm for Muc2 solutions. The low-*q* upturn of the SANS intensity profile of the gelled mucin solutions is thought to arise from the complexation and aggregation of mucin molecules. This can be described as the sum of two contributions [Eq. (2)], as shown in Figures 5c and 5d. This also allowed us to calculate the zero-*q* extrapolated excess scattering intensity [*I*_ex_(0)] and the average thickness of the microphase-separated aggregates (Ξ) as a function of mucin concentration for both types of mucins at pH 1.4, as shown in Figure 5.

## DISCUSSION

Using microrheology data obtained from purified soluble Muc5ac and Muc2 mucin solutions, we were previously able to identify three distinct regimes in the scaling of the viscoelasticity of mucin solutions with concentration. Briefly at pH 7, up to a critical concentration (c*, c* < 1 mg/ml), mucin oligomers diffuse in the solvent with little interaction with one another. The solutions are viscous and have little elasticity. Up to a second critical concentration, the entanglement concentration *c*_e_, mucins starts to overlap to form a semidilute solution (25 mg/ml for Muc5ac and 30 mg/ml for Muc2). Above *c*_e_, the solution is said to be in the semidilute entangled regime, in which the mucin backbones entangle and reptate, leading to a rapid increase in viscosity and elastic shear modulus as a function of concentration.^11^ At pH 1.4, a significant increase in both the elasticity and viscosity of the solutions is observed in both semidilute and entangled solutions due to a process of pH-induced gelation, compared with their values at pH 7.

The flexible nature of mucin oligomers is observed in our SANS experiments using Kratky plots [*q*^2^*I*(*q*) vs. *q*], as shown in Supporting Information Figure S1. The plateau of the *q*^2^*I*(*q*) curve up to the entanglement concentration (semidilute overlap concentration) of the molecule allowed us to estimate the persistence length of the molecule to be approximately 8–10 nm at both pH 7 and pH 1.4. This is considerably smaller when compared with the overall length of the fibers deduced from X-ray Guinier plots, but comparable with the size of the globular end groups, thus it is concluded that the molecules are relatively flexible.^9^ Additionally, previous structural investigations of the molecule have concluded its flexible nature by measuring the persistence length (found to be 8 nm) and by following Zimm dynamics in DLS experiments (Γ has a *q*^3^ dependence).^17^ The aforementioned observation is in line with the assumption that the protein backbone is fairly flexible with relatively rigid globular regions occurring at each end. Previous transmission electron microscopy studies of ocular mucins have also indicated the flexible behavior of these molecules.^18^ The correlation length (ξ) of the mucins appears to be relatively independent of the pH of solutions in the SANS measurements, which is of the order of 1–2 nm in both cases and likely reflect the correlations between the attached carbohydrates.

SAXS experiments of Muc5ac solutions allowed us to calculate the radius of gyration of the molecule at pH 7 and at pH 3.5. At pH 7, the concentration normalized scattering profiles follow the same overall shape, suggesting that no conformational changes occur up to 20 mg/ml, which is slightly below the entanglement concentration. The Guinier plot of the lowest concentration allowed us to calculate the radius of gyration of the molecule, which was found to be 42 ± 4 nm. As previously reported by our group, secreted mucin oligomers are not strongly associating at low concentrations,^11^ thus explaining the discrepancy between our measurements and previous measurements using AFM with chemically fixed samples (fibers ranging from 200 to 600 nm were observed).^2^ Additionally, the presence of an insoluble and a soluble component of Muc2 in small intestinal mucus was identified, where the insoluble component cannot be isolated without the use of reducing or chaotropic agents.^19^ The absence of chaotropic agents from our isolation protocol excludes the insoluble fraction of mucin either partially or completely from our preparations, whereas the absence of reducing agents ensures that the polymeric properties of the mucin are retained. This can potentially bias the mucin chains used in this study toward smaller sizes when compared with the highly entangled insoluble fraction, without nevertheless compromising their polymeric nature. In addition to the above, two layers of adherent mucus have been identified in the gastrointestinal tract, a loose outer layer and a tighter inner layer.^20^ By not reducing disulfide bonds during purification, the possibility exists that our mucin preparation is enriched in mucins from the looser, outer layer. Nevertheless, the chains exhibit the expected molecular weight range measured previously for such mucins (2–200 MDa for both types of mucin; Supporting Information Figure S3) prepared under low temperatures using proteinase inhibitors to avoid cleavage of the macromolecules and have been shown to possess the associating properties of native mucins.^11^ Our SAXS measurements are also in good agreement with previous measurements of the radius of gyration of porcine gastric Muc5ac using similar high-resolution techniques such as SANS and DLS.^7^,^21^

Previous AFM experiments using deglycosylated PGM showed the significance of the attached carbohydrate side chains to the overall conformation of the molecule, since upon deglycosylation, the molecule collapsed and its overall length was reduced due to the formation of compact globular structures at the deglycosylated regions when compared with its glycosylated counterpart.^22^ The aggregation of mucin fibers on reduction of the pH was also reported, which was not significantly affected by the number of carbohydrates attached to the peptide backbone. Furthermore, the isoelectric point of PGM was found to lie between pH 2 and 3.^2^ At pH 3.5, the radius of gyration of the molecules we measured with a SAXS Guinier plot was found to decrease from 42 ± 4 nm to 23 ± 3 nm. As previously suggested, the overall conformation of the mucin molecules and network is an intricate interplay between the electrostatic repulsion of the attached carbohydrates with the hydrophobic attraction of the bare protein core.^23^,^24^ As the pH of the solution approaches the value of 2 (the p*K*_a_ value of sialic acid is 2), the net charge of the attached carbohydrates is screened, and thus, the repulsive electrostatic forces are diminished. This would lead the molecule to behave more like a neutral polymer, rather than a charged polyelectrolyte, causing a reduction in the size of the molecule.^25^ As with Muc5ac at pH 7, the radius of gyration of the measurement at pH 3.5 was performed using a low-concentration solution, which allowed us to study the effect of pH on the mucin oligomers, rather than the self-assembled mucin polymer in its entirety, which is expected to form crosslinks and cause a dramatic increase in its size. Our data are in good agreement with previous reports of the persistence length of mucin molecules (8 nm), and this flexible nature of the molecule has previously been studied in a range of biophysical experiments.^17^,^23^,^24^ The radius of gyration measured using SAXS experiments at pH 7 is in good agreement with previous DLS experiments. However, DLS results indicate that the mucin molecules increase in size at low pH in contradiction with the current study.^17^ The contraction of the molecule at pH 3.5 was measured using SAXS experiments and can be explained by the screening of the charges of the attached side chains and the eventual domination of the forces exerted by the associating hydrophobic regions. SAXS measurements have the advantage over DLS that they are less sensitive to the slow modes that often occur in polyelectrolyte solutions at low polymer and salt concentrations. Another possible explanation is that the oligosaccharide profile of the mucins in the current study is different when compared with mucins used in the study reported by Cao et al. (e.g., a higher charge fraction in our specimens). The carbohydrate composition of mucins can be influenced by a range of factors, for example, the resident microflora and diet of the organism.

The aggregation of the mucin oligomers to form a gel at neutral and acidic pH was further investigated using small-angle neutron scattering, which allowed us to observe the formation of large-scale structures in the low-*q* regime. The concentration-normalized neutron scattering profiles of Muc5ac and Muc2 solutions at pH 7 were of comparable overall shape, which could be described by a single power law over the available *q*-range. This Porod exponent was found to be *n* ∼ 1.7 in the majority of the solutions examined, which corresponds to scattering from mass fractals from linear swollen polymers.^26^ Above the entanglement concentration for the two molecules (∼25 mg/ml for Muc5ac and ∼30 mg/ml for Muc2), there is a second Porod exponent observed in the low-*q* region, which for our samples lies in the region of 2.2 < *n* < 2.3. This transition occurs at *q* ∼ 0.02–0.03 Å, which corresponds to distances of approximately 20–30 nm in real space. Furthermore, there is a slight increase in scattering intensity at low *q*. This observation is in good agreement with our previous microrheology data, which suggested the transition of the mucin solutions from purely viscous, Newtonian liquids to viscoelastic liquids above this entanglement concentration at pH 7. The increased scattering at low *q* could be the result of the complexation of the backbones of the peptide or, as previously suggested, from branching of the termini^14^,^26^ perhaps into the trigonal structures indicated by AFM and TEM measurements.^14^

An inherent limitation of scattering experiments with gels is that fairly featureless scattering profiles are obtained from these polydisperse systems, which limit the information one can extract from them. A minimal model of Porod exponents was used to infer the conformational changes occurring in the mucin gel at low pH, as more complicated models incorporating globular domains would require overfitting of the data.^9^

With respect to the hexagonal lamellar model for MUC2 mucin,^14^ the scattering data provide a series of contradictions. First, there should be a diffraction peak in both SANS and SAXS experiments at 15 nm from the hexagonal interlayer ordering. No such diffraction peak is observed in our experiments. Furthermore, for stacked membranes, there should be an additional smectic-like reflection in the SANS and SAXS experiments due to the interlamellar spacing. Again, no such reflection is observed in the data. In contrast to the membrane model, the SANS/SAXS data are well fit by a standard model for a disorganized gel with liquid-like structuring. It is possible that our method of mucin extraction has enriched the pool of mucins investigated with mucins originating from the outer mucus layer, which do not exhibit the same associative properties of mucins during synthesis as the high-density storage state that exists in mucin granules.^16^ Previous particle-tracking data at low concentrations performed by our group indicated a Fuoss law at lower concentrations (viscosity scales as the square root of the polymer concentration^11^), which also points to liquid-like polymer arrangements.

An important property of gastric mucus is its ability to form pH-switchable gels at low pH, which is a defense mechanism of the body against the hostile environment of the stomach during active digestion. The suggested model for the pH-switchable gelation of gastric mucin involves the complexation of hydrophobic regions in the nonglycosylated regions of the molecules, which are exposed at low pH.^2^ These hydrophobic regions have von-Willebrand-like D-domains at the two termini of the molecule, which are stabilized by salt bridges at neutral pH between negatively charged carboxylates and positively charged amino acids. The protonation of these carboxylates at low pH breaks the stabilizing salt bridges, exposing the hydrophobic domains, which then associate with the equivalent regions of neighboring molecules and act as crosslinks between mucins, causing gelation.^11^,^27^ Previous reports on the pH response of the porcine gastric mucins suggested that the mucin molecule has a net charge of zero at pH 2, a negative charge above this pH and possibly a positive charge below this pH value.^23^,^24^ Furthermore, a process of liquid–liquid phase separation is expected at the lower extreme of the pH range, and it can be macroscopically observed. In the experiments reported here, no macroscopic phase separation was identified when visually inspecting the samples, and thus, we deduce that a relatively uniform process of gelation has occurred.

The SANS intensity profiles of the mucin solutions at pH 1.4 exhibit a large increase in the scattering intensity in the low-*q* regime when compared with the intensity profiles of the mucin solutions of the same concentration at pH 7. Excess scattering in the low-*q* range most likely corresponds to scattering from larger length-scale structures within the solution because of the complexation of the exposed hydrophobic regions at the end termini of the molecules. Furthermore, the concentration-normalized scattering profiles of each type of mucins overlap with themselves, suggesting a common gelling mechanism throughout the concentration range examined for each of the mucins. There are two distinct regions identified. First, there is an exaggerated tail in the low-*q* regime with Porod exponent *n* = 3 for Muc5ac and *n* = 4 for Muc2. Second, there is a transition to a *n* = ∼1.7 Porod exponent in the intermediate *q*-range, with the transition occurring at *q* ∼ 0.02 Å. The different Porod exponents of the two types of molecules indicate that Muc2 undergoes a more complete process of micro/nano phase separation during gelation than Muc5ac; *n* = 3 corresponds to scattering from rough surfaces, whereas *n* = 4 from smooth surface fractals. This difference could be attributed to the presence of more hydrophobic cysteine-rich domains (D-domains) in the protein backbone of the Muc5ac molecule than Muc2; the more even distribution of associating hydrophobic domains along the Muc5ac molecule could potentially lead to a different morphology in the crosslinked gel network.

For us to characterize this process of phase separation, the overall scattering profile of the gelled solutions at pH 1.4 was fitted with Eq. (2), which decomposes the overall *I*(*q*) into two contributions: the Ornstein-Zernicke equation, which describes the scattering from semidilute polymer solutions, and the Debye-Bueche term, which describes a two-phase structure with a sharp boundary. Figure 5 shows the extracted amplitude of heterogeneity *I*_ex_(0), microphase-separated aggregate size Ξ and the semidilute correlation length *ξ*. The semidilute correlation length calculated from the Ornstein-Zernicke equation was of the same order as previously measured for pH neutral solutions, ∼2 nm for Muc5ac and approximately 1–1.5 nm for Muc2. These numbers remain largely unchanged upon the reduction of the pH of the solution, suggesting that there are no significant structural differences between the ungelled and gelled mucin solutions at this length scale. The most probable origin for the semidilute scattering contributions would be correlations between the attached carbohydrates along the peptide backbone, which are not believed to take part in the gel-forming mechanism and are not expected to be particularly sensitive to a change in concentration.^22^ The microphase separation length Ξ was found to monotonically increase with concentration as expected for microphase-separated hydrogels. The increase of the microphase separation [*I*_ex_(0)] and the semidilute intensity [*I*_1_(0)] terms with concentration was previously observed with randomly charged linear ionomers at concentrations above their gelling point.^28^

The distinction between the biochemical, physical, and physiological characteristics of the constituent mucin molecules and the properties of the resulting polymer network is crucial. The properties of a gel cannot yet be adequately predicted from the properties of the individual polymer components, even more so when dealing with these incredibly large and complex biomolecules.^15^ The vast majority of previous reports on mucin structure were performed after the use of fixative agents, which could potentially induce chemical crosslinking between the molecules and induce artifacts in data analysis. Various different chemically fixed granules have produced EM sections, which resemble interconnected, polygon-like structures, each containing very different biopolymers networks, but failed to reproduce this result without the use of fixative agents.^16^ A lot of good quality research has been conducted in the past decade regarding the building blocks of the polymer network of the mucus observed individually; however, very little has been done to look at the self-assembly of mucins in their network structure. We therefore hope that our work provides some new insights into the structure and dynamics of the mucin network using some robust biophysical techniques with biochemically relatively well-defined mucins.

## CONCLUSION

The concentration scaling and pH-switchable gelation of purified pig gastric and duodenal mucin solutions were investigated using small-angle X-ray and neutron scattering. This work is an extension of our previously reported microrheology investigations, which identified three distinct regimes in the scaling of the viscoelasticity of mucin with concentration. In the dilute regime at pH 7, mucins exist as diffusing, noninteracting oligomers in solution, with no contribution to the macroscopic elastic properties of the solution. Above a critical concentration at pH 7, c*, mucins start to overlap and fibrous aggregation occurs, increasing the end-to-end size of the chains in the semidilute unentangled regime. Both Muc5ac and Muc2 mucins were demonstrated to follow the same overall conformation in this regime based on the scattering profiles. Above a second critical concentration at pH 7, the entanglement concentration (*c*_e_), the mucin backbone is expected to entangle and reptate, with a sharp increase in viscosity. The polymer network was observed to undergo a conformational change at around the entanglement concentration, as observed from Porod plots constructed using SANS data, from *q*^−1.7^ to *q*^−2.25^.

Kratky plots of SANS data indicate that the persistence lengths of Muc5ac and Muc2 are similar at 8 ± 2 nm. The chains are thus seen to be fairly flexible, and the persistence length is relatively independent of pH.

The internal structure of mucin hydrogels at pH 1.4 was investigated using SANS experiments, and their structure was similar to that observed previously with synthetic hydrogels. There was considerable excess scattering in the low-*q* regime of the scattering profiles obtained at low pH when compared with their pH 7 counterparts, which indicated the formation of large-scale heterogeneities as a result of crosslinking. This microphase separation process was successfully described by decomposing the overall scattering profile into two contributions, which revealed an increase in the size of the phase-separated aggregates with increasing concentration.

## MATERIALS AND METHODS

### Mucin Purification

Porcine gastrointestinal mucins were used as a model for human mucins. Soluble mucins were extracted from collected mucus as previously described.^11^ Briefly, the mucus layer was scraped from the epithelium of freshly collected pig stomachs and small intestines, homogenized in a 0.2*M* NaCl buffer containing protease inhibitors and 0.2% w/w sodium azide, followed by two steps of CsCl density gradient centrifugation. The mucin-rich fractions were pooled, extensively dialyzed against deionized H_2_O and lyophilized. Purified mucin solutions were prepared by dissolving mucin in 10 m*M* phosphate buffer, where the pH was adjusted using aqueous HCl. All the solutions were prepared 48 h prior to the experiments so that enough time was allowed for them to equilibrate.^7^,^29^,^30^

### Small-Angle X-ray Scattering Experiments

Mucin solutions at two different pHs (3.5 and 7) were investigated at varying protein concentrations using SAXS experiments. The experiments were performed at the high-brilliance ID02 beamline of the European Synchrotron Radiation Facility in Grenoble, France. The sample to detector distance was a combination of distances (1 and 5 m) to cover a wide range of wave vectors *q*, spanning from 2 × 10^−2^ to 10 nm^−1^. *q* is defined as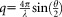, where *θ* is the scattering angle and *λ* is the X-ray wavelength (0.1 nm). The measurements were performed in a flow-through capillary of 2 mm diameter to ensure an accurate subtraction of the background (buffer solution) and negligible beam damage of the samples. The two-dimensional scattering patterns were recorded using a fiber-optic-coupled CCD device (FreLon) and were normalized to an absolute intensity scale after applying the detector corrections for spatial homogeneity and linearity. Normalized SAXS patterns were azimuthally averaged to obtain the one-dimensional scattering profiles [*I*(*q*) vs. *q*].

### Small-Angle Neutron Scattering Experiments

SANS measurements were performed at the SANS2D diffractometer at the ISIS Facility, Rutherford Appleton Laboratory, UK. SANS2D is a time-of-flight small-angle scattering instrument, which uses neutrons with an incident wavelength of 1.75 < *λ* < 16.5 Å.^31^ The accessible *q*-range using the 1 m^2^ detector at 4 m distance was in the range 0.005–0.59 Å^−1^. Raw data were corrected for wavelength dependence of measured sample transmissions, incident spectrum, and detector efficiencies and placed on an absolute scale *I*(*q*) (cm^−1^) by comparison with scattering from a partially deuterated polystyrene standard.^32^,^33^ The use of D_2_O solvent provided contrast between the scatterers and the solvent. The pH* (measured pH using H_2_O-calibrated strips) of the solutions was measured using Whatman CF Indicator pH strips and then subsequently converted to equivalent pH,^34^ that is, the pH was recalibrated for the presence of D_2_O. Quartz Hellma cells (2 mm path length) were used for measurements, with an incident beam of diameter of 8 mm. The scattering profile of the solvent and the empty cell were measured and subsequently subtracted from the raw SANS data so that the normalized scattering data from the solution was obtained.

### Scattering Data Analysis

The scattering intensity of the semidilute polymeric solutions can be described using the Ornstein-Zernicke equation, which is defined as follows:


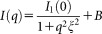
(1)

where *I*_1_(*O*) is the extrapolated intensity where *q* tends to zero, *ξ* is the correlation length, and *B* is the incoherent and inelastic background scattering. This expression is based on the random-phase approximation to describe single-phase systems with liquid-like ordering.^35^,^36^ It is empirically found to be a good fit to SANS data from semidilute polymeric solutions.^37^ *ξ* can describe both interchain and intercluster correlations (mesh sizes). For nanophase-separated systems, Eq. (1) needs to be modified to describe the excess scattering at low *q* due to the phase-separated morphology. A Debye-Bueche term is typically added to describe the excess intensity at low *q* in gel structures:


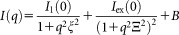
(2)

where *I*_ex_(0) represents the extrapolated zero-*q* intensity of the low-*q* intensity profile, which arises from larger scale structures within the solution, and Ξ is the average thickness of the nanophase-separated aggregates.^28^

Furthermore, by constructing Porod plots {log[*I*(*q*)] vs. log(*q*)} from SANS data and by fitting a function of the form,


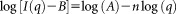
(3)

where *A* is a constant and *B* is the background scattering, we were able to calculate *n*, the Porod exponent related to the fractal dimension of the scattering objects (6 − *n*). A Porod exponent *n* = 1 is expected from scattering from rigid rods, whereas *n* = 4 represents scattering from smooth three-dimensional interfaces, as indicated by the phase boundaries associated with Debye-Beuche scattering in Eq. (2). A Porod slope in the range 3 < *n* < 4 is expected from rough interfaces called surface fractals. *n* = 2 is expected from the intermediate *q*-range of random Gaussian coils, approximately true for gels described by Eq. (1) and from perfectly flat sheets. *n* = 5/3 is found for fully swollen polymers in good solvent. A slope in the range 2 < *n* < 3 is for mass fractals such as polymeric networks.^38^

To extract the radius of gyration of the mucin molecules, Guinier plots were constructed by plotting ln[I(*q*)] vs. *q*^2^. By fitting the Guinier approximation,


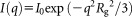
(4)

the radius of gyration (*R*_g_) was calculated. Fits were limited to the initial part of the scattering curves, where the inequality *qR*_g_ < 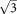 is satisfied.^39^
