# Supplementary figures and images for: A Combined Small-Angle X-ray and Neutron Scattering Study of the Structure of Purified Soluble Gastrointestinal Mucins
Source: Biopolymers. 2014 Jul 11;101(12):1154–64. doi: 10.1002/bip.22523 (PMC4654235; doi:10.1002/bip.22523)

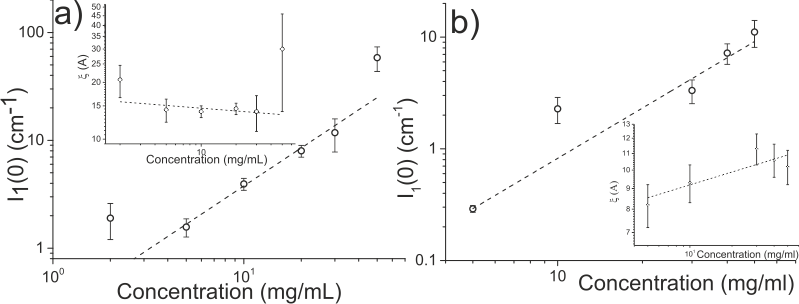

Supplement: Supplementary file 2 — Supplementary Information Figure 2. [file bip0101-1154-sd2.tif]

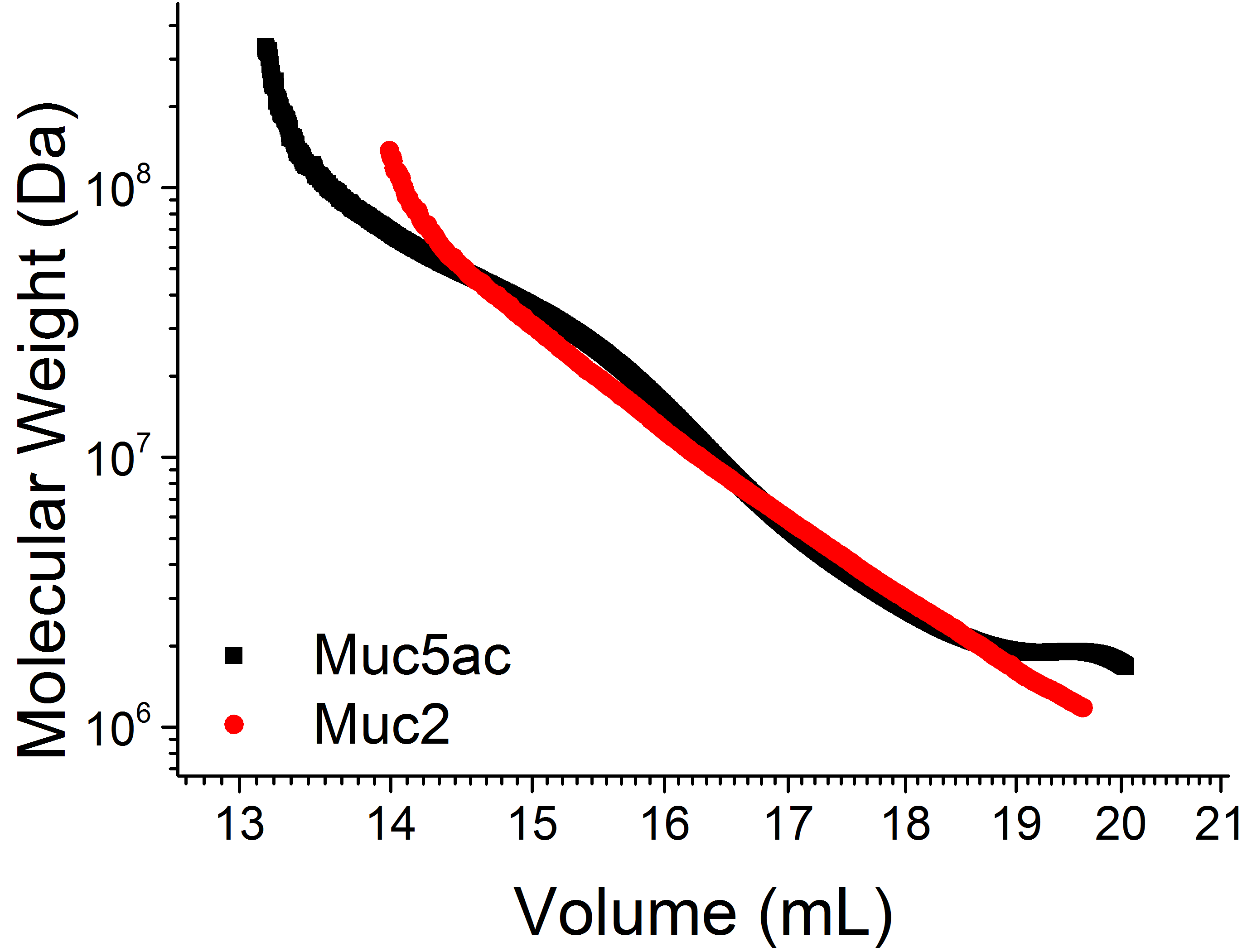

Supplement: Supplementary file 3 — Supplementary Information Figure 3. [file bip0101-1154-sd3.tif]
